# Supplementary material for: Generative AI mitigates representation bias and improves model fairness through synthetic health data
Source: PLoS Comput Biol. 2025 May 19;21(5):e1013080. doi: 10.1371/journal.pcbi.1013080 (PMC12112403; doi:10.1371/journal.pcbi.1013080)
Supplement: S1 Appendix — (PDF) [file pcbi.1013080.s001.pdf]

# S1 Appendix: Distribution Plots for Sepsis

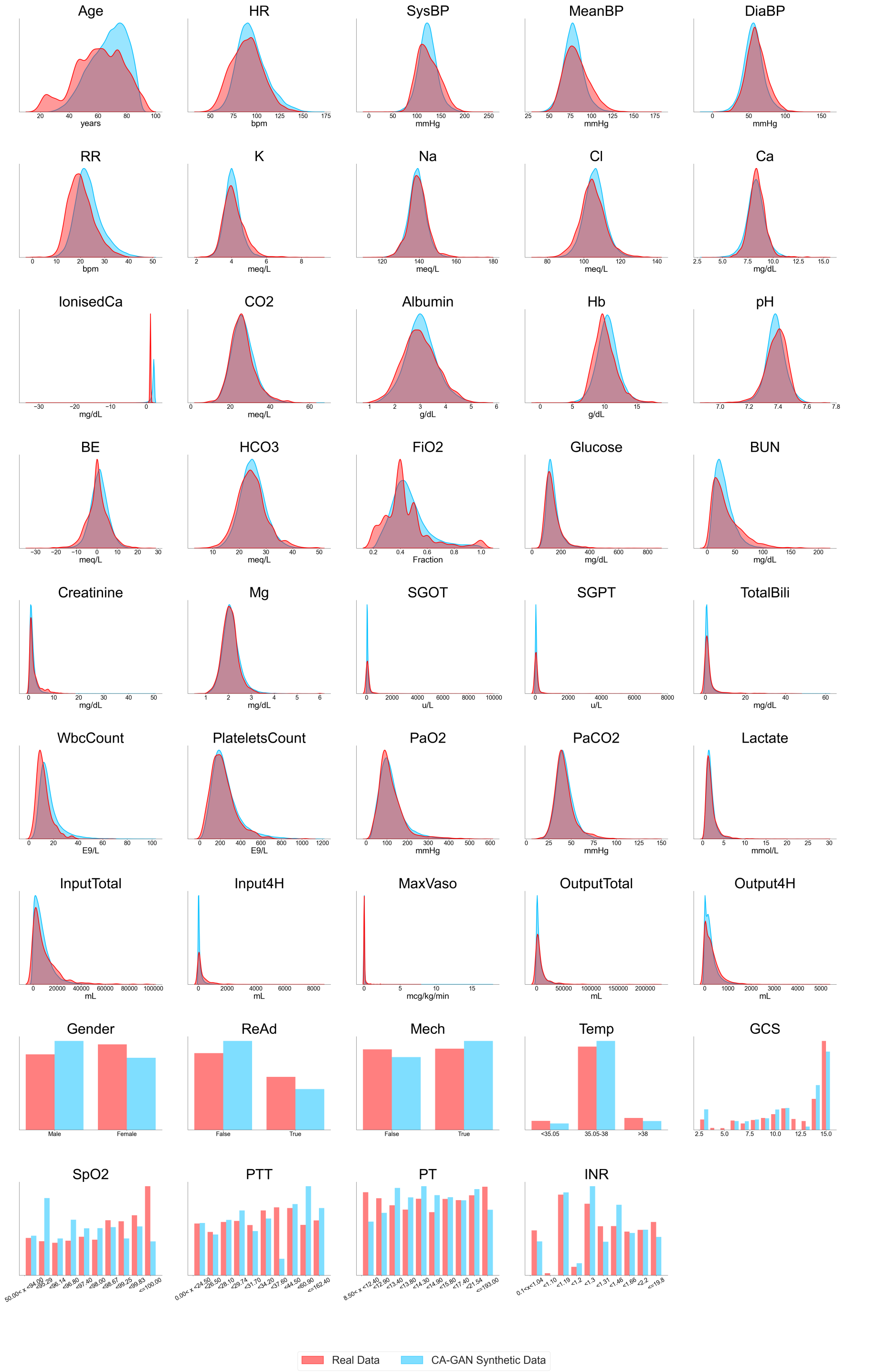


**Fig. A**: Overlaid distribution plots of real data and CA-GAN synthetic data for each variable in the sepsis dataset.

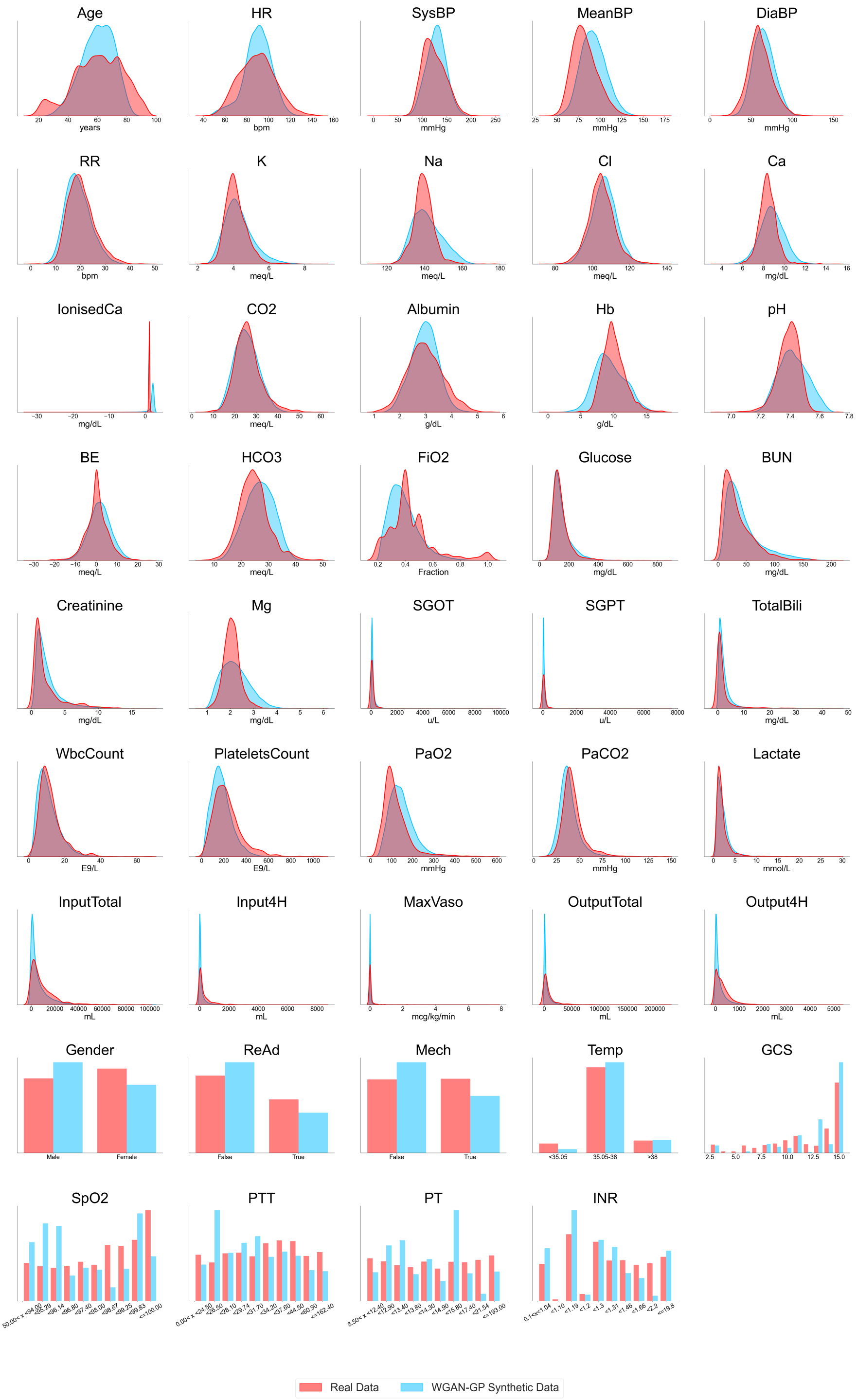


**Fig. B**: Overlaid distribution plots of real data and WGAN-GP* synthetic data for each variable in the sepsis dataset.

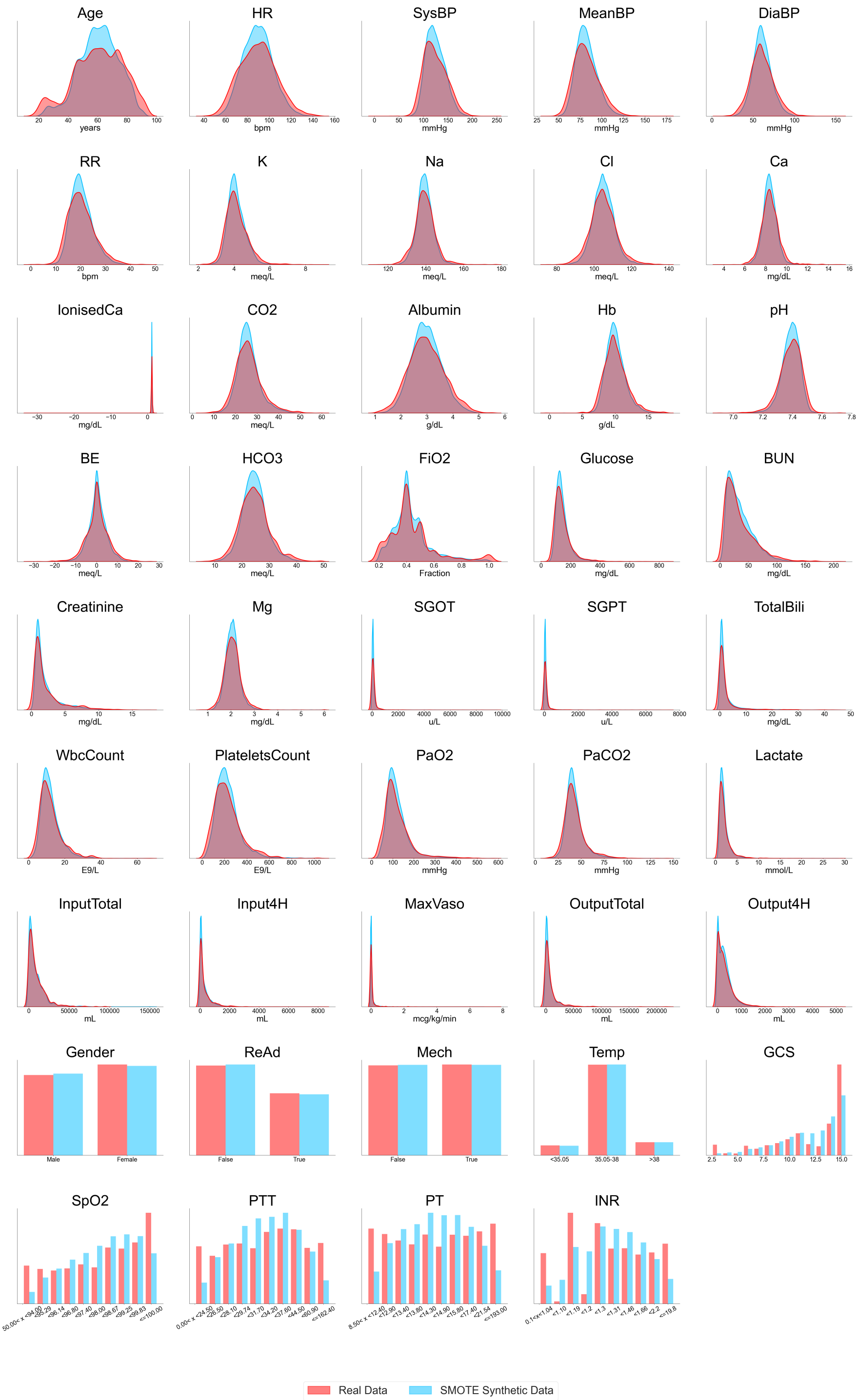


**Fig. C**: Overlaid distribution plots of real data and SMOTE synthetic data for each variable in the sepsis dataset.
