# Supplementary material for: Generative AI mitigates representation bias and improves model fairness through synthetic health data
Source: PLoS Comput Biol. 2025 May 19;21(5):e1013080. doi: 10.1371/journal.pcbi.1013080 (PMC12112403; doi:10.1371/journal.pcbi.1013080)
Supplement: S2 Appendix — (PDF) [file pcbi.1013080.s002.pdf]

S2 Appendix: Datasets

Table A: Variables in the acute hypotension dataset. For each variable, the data type, the unit in which it is expressed, and the distribution statistics are presented.

Variable Name	Data Type	Unit	Descriptive Statistics	
Mean Arterial Pressure	numeric	mmHg	Median: 68.00 (Q1: 61.00, Q3: 76.00)	
Diastolic Blood Pressure	numeric	mmHg	Median: 54.00 (Q1: 47.00, Q3: 62.00)	
Systolic BP	numeric	mmHg	Median: 107.00 (Q1: 96.00, Q3: 120.00)	
Urine	numeric	mL	Median: 75.00 (Q1: 45.00, Q3: 140.00)	
Alanine Aminotransferase	numeric	IU/L	Median: 35.00 (Q1: 23.00, Q3: 35.00)	
Aspartate Aminotransferase	numeric	IU/L	Median: 50.00 (Q1: 33.00, Q3: 50.00)	
Partial Pressure of Oxygen	numeric	mmHg	Median: 102.00 (Q1: 99.00, Q3: 102.00)	
Lactate	numeric	mmol/L	Median: 1.80 (Q1: 1.20, Q3: 1.80)	
Serum Creatinine	numeric	mg/dL	Median: 1.10 (Q1: 0.80, Q3: 1.90)	
Fluid Boluses	categorical	mL	4 Classes	
			[0,250) : 95.75%;	[250,500) : 0.61%
			[500,1000) : 1.73%;	$\geq$ 1000 : 1.91%
Vasopressors	categorical	mcg/kg/min	4 Classes	
			0 : 81.95%;	(0,8.4) : 9.02%
			[8.4,20.28) : 4.51%;	$\geq$ 20.28 : 4.52%
Fraction of Inspired Oxygen	categorical	fraction	10 Classes	
			$\leq$ 0.2 : 0.44%;	0.2 : 0.45%
			0.3 : 5.29%;	0.4 : 15.52%
			0.5 : 61.64%;	0.6 : 4.36%
			0.7 : 2.78%;	0.8 : 1.43%
			0.9 : 1.42%;	1.0 : 6.67%
Glasgow Coma Scale Score	categorical	point	13 Classes	
			3 : 4.57%	4 : 0.72%
			5 : 0.44%	6 : 2.37%
			7 : 3.44%	8 : 4.03%
			9 : 3.63%	10 : 6.61%
			11 : 4.48%	12 : 1.22%
			13 : 3.23%	14 : 11.48%
			15 : 53.80%	
Urine Data Measured (M)	binary	-	False: 55.64%	True: 44.36%
ALT or AST (M)	binary	-	False: 97.73%	True: 2.27%
FiO2 (M)	binary	-	False: 88.80%	True: 11.20%
GCS (M)	binary	-	False: 78.06%	True: 21.94%
PaO2 (M)	binary	-	False: 95.45%	True: 4.55%
Lactic Acid (M)	binary	-	False: 95.61%	True: 4.39%
Serum Creatinine (M)	binary	-	False: 92.79%	True: 7.21%

Table B: Variables in the sepsis dataset. For each variable, the data type, the unit in which it is expressed, and the distribution statistics are presented.

Variable Name	Data Type	Unit	Descriptive Statistics	
Age	numerical	years	Median: 66.95 (Q1: 54.21, Q3: 78.70)	
Heart Rate (HR)	numerical	bpm	Median: 86.75 (Q1: 75.40, Q3: 98.86)	
Systolic BP	numerical	mmHg	Median: 118.17 (Q1: 105.33, Q3: 134.20)	
Mean BP	numerical	mmHg	Median: 77.25 (Q1: 69.12, Q3: 87.00)	
Diastolic BP	numerical	mmHg	Median: 56.81 (Q1: 48.60, Q3: 65.67)	
Respiratory Rate (RR)	numerical	bpm	Median: 20.00 (Q1: 16.86, Q3: 23.83)	
Potassium (K)	numerical	meq/L	Median: 4.00 (Q1: 3.70, Q3: 4.30)	
Sodium (Na)	numerical	meq/L	Median: 139.00 (Q1: 136.00, Q3: 142.00)	
Chloride (Cl)	numerical	meq/L	Median: 105.00 (Q1: 101.00, Q3: 109.00)	
Calcium (Ca)	numerical	mg/dL	Median: 8.30 (Q1: 7.84, Q3: 8.74)	
Ionised Ca	numerical	mg/dL	Median: 1.13 (Q1: 1.08, Q3: 1.18)	
Carbon Dioxide (CO2)	numerical	meq/L	Median: 26.00 (Q1: 22.75, Q3: 29.00)	
Albumin	numerical	g/dL	Median: 2.90 (Q1: 2.50, Q3: 3.40)	
Hemoglobin (Hb)	numerical	g/dL	Median: 10.00 (Q1: 9.04, Q3: 11.20)	
pH	numerical	-	Median: 7.40 (Q1: 7.35, Q3: 7.44)	
Arterial Base Excess	numerical	meq/L	Median: 0.00 (Q1: -2.00, Q3: 3.15)	
Bicarbonate (HCO3)	numerical	meq/L	Median: 25.00 (Q1: 22.00, Q3: 28.00)	
FiO2	numerical	fraction	Median: 0.40 (Q1: 0.40, Q3: 0.50)	
Glucose	numerical	mg/dL	Median: 129.67 (Q1: 109.00, Q3: 157.60)	
Blood Urea Nitrogen	numerical	mg/dL	Median: 24.00 (Q1: 15.00, Q3: 41.00)	
Creatinine	numerical	mg/dL	Median: 1.00 (Q1: 0.70, Q3: 1.60)	
Magnesium (Mg)	numerical	mg/dL	Median: 2.03 (Q1: 1.90, Q3: 2.27)	
SGOT	numerical	u/L	Median: 41.00 (Q1: 25.00, Q3: 86.00)	
SGPT	numerical	u/L	Median: 32.00 (Q1: 18.00, Q3: 69.00)	
Total Bilirubin	numerical	mg/dL	Median: 0.70 (Q1: 0.40, Q3: 1.80)	
White Blood Cell Count	numerical	E9/L	Median: 11.20 (Q1: 8.20, Q3: 15.20)	
Platelets Count	numerical	E9/L	Median: 207.85 (Q1: 141.00, Q3: 296.00)	
paO2	numerical	mmHg	Median: 105.25 (Q1: 82.57, Q3: 140.00)	
paCO2	numerical	mmHg	Median: 40.22 (Q1: 35.25, Q3: 46.09)	
Lactate	numerical	mmol/L	Median: 1.60 (Q1: 1.10, Q3: 2.30)	
Total Input Fluids	numerical	mL	Median: 6569.08 (Q1: 2540.00, Q3: 13047.66)	
Input 4H	numerical	mL	Median: 80.01 (Q1: 20.34, Q3: 327.50)	
Max Vasopressors in 4H	numerical	mcg/kg/min	Median: 0.0002 (Q1: 0.00, Q3: 0.0017)	
Total Volume Output	numerical	mL	Median: 3893.00 (Q1: 1300.00, Q3: 10070.00)	
Output 4H	numerical	mL	Median: 248.00 (Q1: 105.00, Q3: 460.00)	
Gender	binary	-	Male: 56.46%	Female: 43.54%
Readmission of Patient	binary	-	False: 67.75%	True: 32.25%
Mechanical Ventilation	binary	-	False: 46.89%	True: 53.11%
Temperature (Temp)	categorical	Celsius	3 Classes	
			<35.05: 8.64%	
			35.05-38: 79.65%	
			>38: 11.71%	
GCS	categorical	point	13 Classes	
			3: 5.10%	4: 0.76%
			5: 0.93%	6: 4.51%
			7: 3.72%	8: 4.12%
			9: 5.17%	10: 7.93%
			11: 9.68%	12: 3.98%
			13: 3.45%	14: 13.03%
			15: 37.61%	
Pulse Oximetry Saturation	categorical	%	10 Classes	
			[50.00,94.00): 9.34%	[94.00,95.29): 10.54%
			[95.29,96.14): 10.11%	[96.14,96.80): 9.49%
			[96.80,97.40): 9.77%	[97.40,98.00): 8.70%
			[98.00,98.67): 12.05%	[98.67,99.25): 9.96%
			[99.25,99.83): 9.99%	[99.83,100.00]: 10.05%
Partial Thromboplastin Time	categorical	s	10 Classes	
			[0.00,24.50): 9.69%	[24.50,26.50): 9.92%
			[26.50,28.10): 10.35%	[28.10,29.74): 10.02%
			[29.74,31.70): 9.76%	[31.70,34.20): 10.09%
			[34.20,37.60): 10.08%	[37.60,44.50): 10.04%
			[44.50,60.90): 10.04%	[60.90,162.40]: 9.99%
Prothrombin Time	categorical	s	10 Classes	
			[8.50,12.04): 10.00%	[12.04,12.90): 9.77%
			[12.90,13.40): 9.68%	[13.40,13.80): 9.67%
			[13.80,14.30): 10.54%	[14.30,14.90): 10.03%
			[14.90,15.80): 10.14%	[15.80,17.40): 9.99%
			[17.40,21.54): 10.20%	[21.54,193]: 9.98%
INR	categorical	-	10 Classes	
			[0.10,1.04): 10.00%	[1.04,1.10): 0.48%
			[1.10,1.19): 17.55%	[1.19,1.20): 2.15%
			[1.20,1.30): 16.88%	[1.30,1.31): 11.23%
			[1.31,1.46): 11.68%	[1.46,1.66): 10.02%
			[1.66,2.2): 9.79%	[2.2,19.8]: 10.22%

Data preprocessing

Detailed description of the data preprocessing steps are available from our previous publication [1] in Section 1 of the supplementary material [2]. However, for completeness we highlight some of the main approaches. For the acute hypotension dataset we included adult patients (18 or over) in the MIMIC-III dataset with at least 24 hours of data, aggregating 48 hours of clinical variables from patients with seven or more mean arterial pressure (MAP) values of 65 mmHg or less, indicating acute hypotension. Missing values were replaced with the last available data, while an indicator variable was used to denote whether a value was measured or not. For the sepsis dataset we included adult patients only who had any suspicious infections based on history of administering antibiotics. We included all the variables from at least 44 hours before the suspected infection and up to 28 hours after. The missing data was imputed using nearest neighbour method. More detailed information is available in https://static-content.springer.com/esm/art%3A10.1038%2Fs41597-022-01784-7/MediaObjects/41597_2022_1784_MOESM4_ESM.pdf

[1] Kuo, N.IH., Polizzotto, M.N., Finfer, S. et al. The Health Gym: synthetic health-related datasets for the development of reinforcement learning algorithms. Sci Data 9, 693 (2022). https://doi.org/10.1038/s41597-022-01784-7

[2] https://static-content.springer.com/esm/art%3A10.1038%2Fs41597-022-01784-7/MediaObjects/41597_2022_1784_MOESM4_ESM.pdf
