# Supplementary material for: Generative AI mitigates representation bias and improves model fairness through synthetic health data
Source: PLoS Comput Biol. 2025 May 19;21(5):e1013080. doi: 10.1371/journal.pcbi.1013080 (PMC12112403; doi:10.1371/journal.pcbi.1013080)
Supplement: S3 Appendix — (PDF) [file pcbi.1013080.s003.pdf]

S3 Appendix: UMAP and t-SNE parameters

In this study, we used t-SNE and UMAP algorithms to perform dimensionality reduction on our datasets and highlight the differences between the results of the three methods under analysis. The following parameters were used for each algorithm:

t-SNE:

Library: scikit-learn version 1.2.2
Parameters for sepsis: $n\_components = 2$, $n\_iter = 500$, $learning\_rate = 100$, $perplexity = 50$
Parameters for acute hypotension: $n\_components = 2$, $n\_iter = 100$, $learning\_rate = 1000$, $perplexity = 30$

UMAP:

Library: umap-learn version 0.5.3
Parameters: $n\_neighbors = 5$, $spread = 5$, $min\_dist = 0.5$
