# Supplementary material for: Generative AI mitigates representation bias and improves model fairness through synthetic health data
Source: PLoS Comput Biol. 2025 May 19;21(5):e1013080. doi: 10.1371/journal.pcbi.1013080 (PMC12112403; doi:10.1371/journal.pcbi.1013080)
Supplement: S4 Appendix — (PDF) [file pcbi.1013080.s004.pdf]

S4 Appendix: Joint distributions of variables

We have carried out an analysis of a set of variables that are clinically known to follow a joint distribution, namely systolic, diastolic and mean arterial blood pressure. This was to investigate whether CA-GAN can capture joint distributions of variables and whether synthetic data are clinically meaningful, where we know that systolic blood pressure is always higher than diastolic blood pressure. Using a scatter plot in Figure A we show that the joint distribution of real data is similar to that of synthetic data.

Following from this, we have also implemented several sanity checks based on clinical knowledge to ensure that the generated synthetic data is clinically meaningful. In this respect, we have performed checks to investigate whether systolic BP values are always lower than diastolic. From our analysis, in the real sepsis dataset, 99.94% of values of these variables were correct; that is, systolic values were always lower than the diastolic values. In the synthetic sepsis dataset, this figure was 99.93%, with only 0.01% difference between the real and the synthetic dataset. On the other hand for the hypotension dataset there were 99.86% correct values of systolic and diastolic variables versus 99.84% in the synthetic dataset, representing a 0.02% difference. This analysis suggests that our architecture captures the structure of the real data quite well.

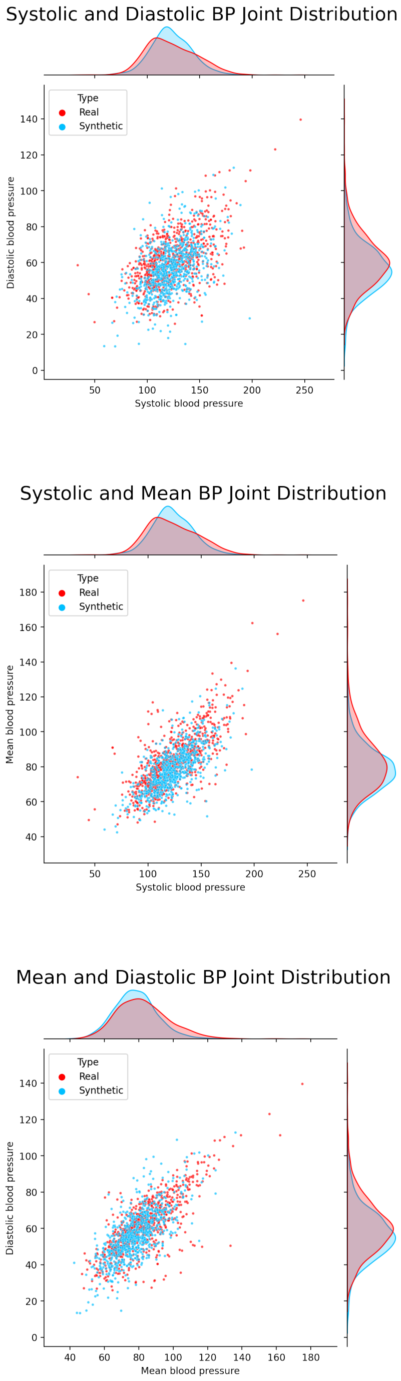

Fig. A: Joint distribution plot of real sepsis data and CA-GAN synthetic data for the variables *Systolic blood pressure*, *Diastolic blood pressure* and *Mean arterial pressure*.
