# Supplementary material for: Generative AI mitigates representation bias and improves model fairness through synthetic health data
Source: PLoS Comput Biol. 2025 May 19;21(5):e1013080. doi: 10.1371/journal.pcbi.1013080 (PMC12112403; doi:10.1371/journal.pcbi.1013080)
Supplement: S5 Appendix — (PDF) [file pcbi.1013080.s005.pdf]

S5 Appendix: Summary of distance metrics

Table A: Statistics for KL-Divergence and Maximum Mean Discrepancy between the distribution of real and synthetic data.

	KL-divergence			MMD		
	SMOTE	WGAN-GP*	**CA-GAN**	SMOTE	WGAN-GP*	**CA-GAN**
MEDIAN	0.03407	0.02711	0.00629	0.00752	0.00217	0.00089
MEAN	0.05754	0.13518	0.14128	0.01500	0.05861	0.04790
STD DEV	0.07222	0.22330	0.31578	0.02385	0.09311	0.08457
MAX	0.28191	0.91622	1.36841	0.09954	0.25091	0.24806
MIN	0.00068	0.00010	0.00005	0.00047	0.00001	0.00001

(a) Acute hypotension data

	KL-divergence			MMD		
	SMOTE	WGAN-GP*	**CA-GAN**	SMOTE	WGAN-GP*	**CA-GAN**
MEDIAN	0.02296	0.06105	0.02292	0.00349	0.01299	0.00377
MEAN	0.09946	0.09282	0.06656	0.00583	0.03848	0.02505
STD DEV	0.29030	0.09704	0.08906	0.00702	0.13482	0.11603
MAX	1.88754	0.33564	0.39555	0.02948	0.90096	0.77536
MIN	0.00035	0.00240	0.00199	0.00002	0.00041	0.00003

(b) Sepsis data
