# Supplementary material for: Generative AI mitigates representation bias and improves model fairness through synthetic health data
Source: PLoS Comput Biol. 2025 May 19;21(5):e1013080. doi: 10.1371/journal.pcbi.1013080 (PMC12112403; doi:10.1371/journal.pcbi.1013080)
Supplement: S6 Appendix — (PDF) [file pcbi.1013080.s006.pdf]

S6 Appendix: Description of Downstream Regression Task

For this task, the BiLSTM is trained on the first 20 hours for hypotension and 10 hours for sepsis of the patients' values to predict the next hour, using a sliding window approach. To ensure the fairness of our result, 15% of the time series data points of Black patients and a proportional representation of different genders were set apart as a test set. To account for the stochasticity of the models, we generated five synthetic datasets with CA-GAN. Then, we trained 5 BiLSTM models on each dataset for a total of 25 BiLSTM models (5x(5 per dataset)). The predictions of the trained models were then compared with the test dataset, and the resulting error was averaged across the five models and, subsequently, the five datasets.

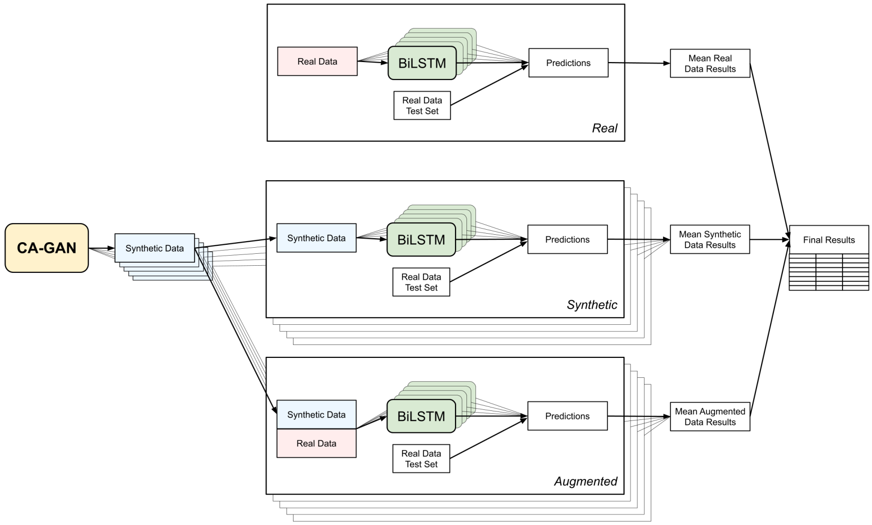

Fig. A: Diagram of BiLSTM Model Training for Continuous Variable Prediction.
