# Supplementary material for: Generative AI mitigates representation bias and improves model fairness through synthetic health data
Source: PLoS Comput Biol. 2025 May 19;21(5):e1013080. doi: 10.1371/journal.pcbi.1013080 (PMC12112403; doi:10.1371/journal.pcbi.1013080)
Supplement: S7 Appendix — (PDF) [file pcbi.1013080.s007.pdf]

S7 Appendix: UMAP Plots

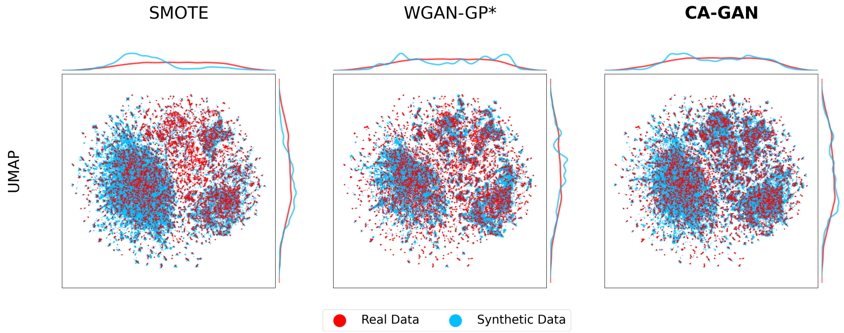

Fig. A: UMAP two-dimensional representations of the acute hypotension dataset for Black patients.

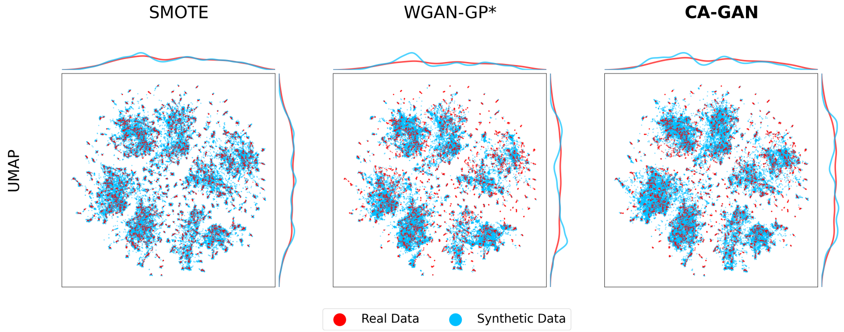

Fig. B: UMAP two-dimensional representations of the sepsis dataset for Black patients.
