# Supplementary material for: Generative AI mitigates representation bias and improves model fairness through synthetic health data
Source: PLoS Comput Biol. 2025 May 19;21(5):e1013080. doi: 10.1371/journal.pcbi.1013080 (PMC12112403; doi:10.1371/journal.pcbi.1013080)
Supplement: S8 Appendix — (PDF) [file pcbi.1013080.s008.pdf]

S8 Appendix: Absolute Differences in Correlations

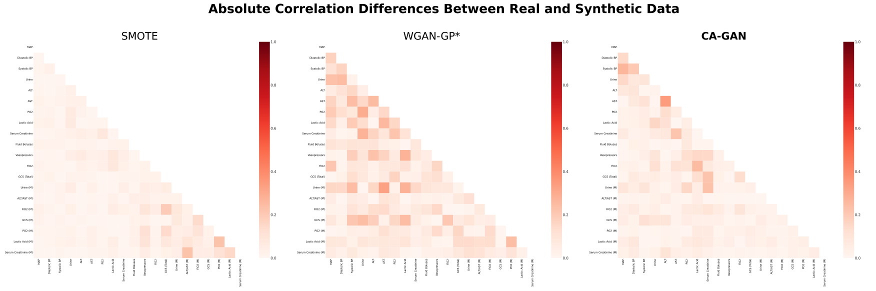

(a) Acute hypotension data

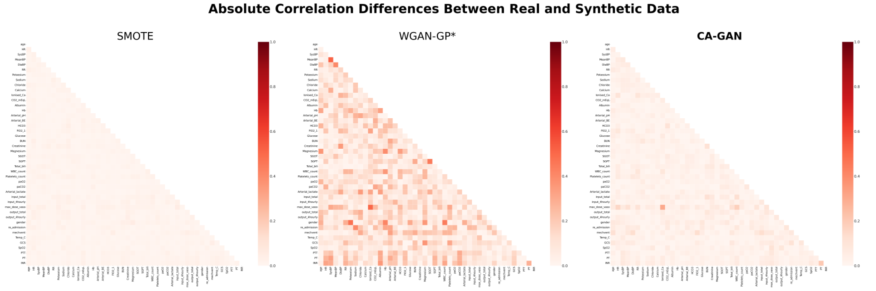

(b) Sepsis data

Fig. A: Absolute difference in correlations between real data and the synthetic data generated with SMOTE, WGAN-GP*, and CA-GAN.
